# The descriptive epidemiology of delirium symptoms in a large population-based cohort study: results from the Medical Research Council Cognitive Function and Ageing Study (MRC CFAS)

**DOI:** 10.1186/1471-2318-14-87

**Published:** 2014-07-28

**Authors:** Daniel HJ Davis, Linda E Barnes, Blossom CM Stephan, Alasdair MJ MacLullich, David Meagher, John Copeland, Fiona E Matthews, Carol Brayne

**Affiliations:** 1 Department of Public Health and Primary Care, University of Cambridge, Cambridge, UK; 2 University of Edinburgh Centre for Cognitive Ageing and Cognitive Epidemiology, Edinburgh, UK; 3 Institute of Health and Society, Newcastle University, Newcastle, UK; 4 Graduate Entry Medical School, University of Limerick, Limerick, Ireland; 5 Department of Psychiatry, University of Liverpool, Liverpool, UK; 6 MRC Biostatistics Unit, Cambridge, UK; 7 MRC Unit for Lifelong Health and Ageing, University College London, 33 Bedford Place, London WC1B 5JU, UK

**Keywords:** Delirium, Dementia, Population, Epidemiology, Algorithm diagnosis

## Abstract

**Background:**

In the general population, the epidemiological relationships between delirium and adverse outcomes are not well defined. The aims of this study were to: (1) construct an algorithm for the diagnosis of delirium using the Geriatric Mental State (GMS) examination; (2) test the criterion validity of this algorithm against mortality and dementia risk; (3) report the age-specific prevalence of delirium as determined by this algorithm.

**Methods:**

Participant and informant data in a randomly weighted subsample of the Cognitive Function and Ageing Study were taken from a standardized assessment battery. The algorithmic definition of delirium was based on the DSM-IV classification. Outcomes were: proportional hazard ratios for death; odds ratios of dementia at 2-year follow-up.

**Results:**

Data from 2197 persons (representative of 13,004) were used, median age 77 years, 64% women. Study-defined delirium was associated with a new dementia diagnosis at two years (OR 8.82, 95% CI 2.76 to 28.2) and death (HR 1.28, 95% CI 1.03 to 1.60), even after adjustment for acute illness severity. Similar associations were seen for study-defined subsyndromal delirium. Age-specific prevalence as determined by the algorithm increased with age from 1.8% in the 65-69 year age group to 10.1% in the ≥85 age group (p < 0.01 for trend). For study-defined subsyndromal delirium, age-specific period prevalence ranged from 8.2% (65-69 years) to 36.1% (≥85 years).

**Conclusions:**

These results demonstrate the possibility of constructing an algorithmic diagnosis for study-defined delirium using data from the GMS schedule, with predictive criterion validity for mortality and dementia risk. These are the first population-based analyses able to account prospectively for both illness severity and an earlier study diagnosis of dementia.

## Background

Delirium is a serious neuropsychiatric syndrome presenting with inattention and global changes in cognition [1-3]. Delirium arises as a consequence of a neurological or systemic illness, medications and psychological stress. It is well-recognized that there is a relationship between predisposing (ageing, cognitive impairment) and precipitating (illness severity) factors such that in the setting of multiple (or severe) predisposing factors, fewer (or less severe) precipitating factors are required [4]. Delirium is therefore a sensitive marker of acute illness in vulnerable older people. This association with acute illness has resulted in the vast majority of delirium studies being undertaken in hospital cohorts [5]. However, this introduces selection biases as not all persons with delirium may reach medical attention. In addition, comparisons to pre-morbid cognitive functions are difficult.

In hospital samples, a common finding is that delirium contributes to persistent cognitive deficits, independently of predisposing and precipitating factors [6]. This has also been reported for subsyndromal delirium, where individuals have one or more of the diagnostic features of delirium [7]. Indeed, any examination of the utility of a delirium definition should incorporate criterion validity tests for future dementia. In prospective community cohort studies, hospitalization predicts adverse cognitive outcomes [8-10], though none has been able to specify if delirium is a key determinant. Delirium is also associated with increased mortality [11], and this should be another criterion by which any definition of delirium should be validated.

The point-prevalence of delirium in the community is thought to be low (0.7%, 95% CI 0.5 to 1.0 in the population aged ≥60 years), though this understanding is based on a systematic review identifying only three prevalence estimates in population samples [12]. Furthermore, epidemiological studies may under-estimate acute illness and/or prevalent delirium because people who are unwell are less likely to be interviewed. However, the period-prevalence may be higher. The Gerontological Regional Database (GERDA) study reported that 27% of persons aged 85 and older in the general population with delirium in the previous month [13]. This suggests that whole population samples could potentially investigate delirium more efficiently if stratified subsamples at higher risk for cognitive impairment are more intensively studied.

Delirium is clinically defined by application of a psychiatric reference standard such as the Diagnostic and Statistical Manual (DSM), where the core features are inattention, altered consciousness, cognitive and/or perceptual disturbance, acute and fluctuating change, in relation to a general medical condition. Based on this, there is an opportunity to construct an algorithmic diagnosis for delirium in population-based cohort studies collecting psychiatric symptoms. Such an approach is well-established in dementia, but yet to be systematically applied in delirium, and particularly not in population studies. Accordingly, using data from the population-based Medical Research Council (MRC) Cognitive Function and Ageing Study (CFAS) the aims of this study were to: (1) construct an algorithm for the diagnosis of delirium in population-based studies using the Geriatric Mental State (GMS) examination based on clinical principles; (2) test the predictive criterion validity of this algorithm against mortality and dementia risk; (3) report the age-specific prevalence of delirium as determined by this algorithm.

## Methods

### Population

Data from the MRC Cognitive Function and Ageing Study (CFAS) were used in this secondary analysis. The principal methods for CFAS have previously been reported [14]. In brief, CFAS was a multi-center study, with sampling from four urban (Newcastle, Nottingham, Oxford and Liverpool), and two rural areas (Cambridgeshire and Gwynedd) in the UK. The present report only concerns the five identical sites (excluding Liverpool). Each center had ethical approval from the Local Research Ethics Committee (*Cambridge*: North West Anglia Health Authority Local Research Ethics Committee (Peterborough); Huntingdon Local Research Ethics Committee; Cambridge Local Research Ethics Committee. *Gwynedd*: Gwynedd Hospitals NHS Trust – NWHA Research Ethics Committee (West). *Newcastle*: Newcastle & North Tyneside Health Authority – Joint Ethics Committee; Northumberland and Tyne & Wear Health Authority – Local Research Ethics Committee. *Nottingham*: QMC NHS Trust Ethics Committee; Nottingham University Medical School Ethical Committee; City Hospital Ethics Committee. *Oxford*: Oxfordshire Health Authority: Central Oxford Research Ethics Committee). Family Health Service Authority lists were used as the sampling frame within a defined geographical area, and this specifically included people resident in institutions. Each individual gave written consent to participate in the study.

**Figure 1:**
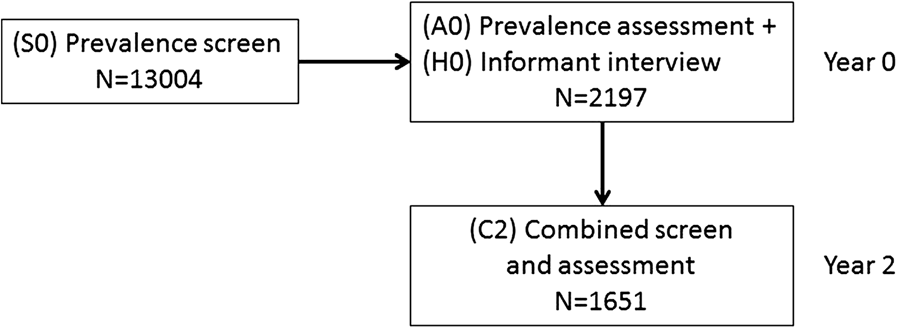
**Assessment and follow-up schedule for the first two years of CFAS.** Schematic showing the numbers assessed, along with informant histories, both at baseline and follow-up. In the text, S0; A0; H0; C2 are described as “Screen”; “Ascertainment”; “Informant”; “Follow-up” respectively.

Figure 1 shows the two stage sampling process for case ascertainment. A screening examination was started in 1991 (“Screen”, n = 13004)^a^. Then, a stratified sample consisted of approximately 20% was selected using center, age (equal numbers aged 65–74 and ≥75), and cognitive ability (weighted toward the more cognitively impaired, based on the screening assessment), and a random subsample from the remaining 80% (“Ascertain”, n = 2640) (mean interval between “Screen” and “Ascertain” was 3 months). Interviews of participants’ nearest informants were also undertaken (“Informant”, n = 2159) (mean interval between “Ascertain” and “Informant” was 3 weeks). Participants were followed at two years, with further subsets examined more frequently, and the whole cohort was re-examined at 6 and 10 years. The number of participants at baseline and at the first two-year follow-up is shown in Figure 1. Mortality outcomes were notified through reports linked to the UK Office of National Statistics.

### Interviews

Interviews were carried out in participants’ usual residence (including if in a care home) by trained interviewers. At screening, information on socio-demographic, physical and behavioral status was collected in addition to health (including self-reported chronic conditions) and cognitive function, assessed using the MMSE. The assessment interview was based on the GMS [15], which was designed to be a structured schedule amenable to administration by non-clinicians. The GMS comprises measures assessing psychiatric symptoms of organicity, depression, anxiety and psychosis, with ratings for each derived using the AGECAT algorithm. The study diagnosis of dementia was based on the GMS B3 AGECAT algorithmic differential diagnosis, where the dementia component is organicity at case level 03 and above. This approach has been validated against formal clinical diagnoses based on DSM-III-R [16]. All information was based on self-report. Informant proxies were also interviewed in a standardized manner using a set of questions complementary to the GMS known as the History and Aetiology Schedule (HAS). Informants were asked questions covering psychiatric symptom clusters occurring in ‘recent weeks and months’. Each interviewer for “Follow-up” undertook assessments blinded to data acquired in the baseline phase.

Questions from “Ascertain” and “Informant” (Figure 1) pertaining to delirium symptoms are shown in Table 1. These were used to define an algorithm for a study definition of delirium based on DSM-IV, where participants were required to demonstrate all three of: (i) acute change; (ii) fluctuation; (iii) inattention and/or drowsiness (Table 2). Subsyndromal delirium was defined as having at least one of these features. In addition, interviewers were asked to judge if responses were affected by their subjective rating of any current acute illness in the participants (categorized as: none, mild, moderate, or severe).

**Table 1:** The prevalence of delirium symptom clusters at baseline

**Symptom**	**Interview question (yes/no)**	**N (2197)**	**%**
Acute change	Has there been sudden worsening in mental confusion in recent weeks or months, which has continued to the present time?	199	9.1%
Fluctuation	Are there episodes lasting days or weeks when his/her thinking seems quite clear and then becomes muddled?	264	12.0%
	Are there long periods during the day when s/he is lucid and not confused (that is, knows where s/he is and knows what s/he is doing and saying)?		
	Does s/he get confused at night, wander about or talk nonsense?		
	Or at any other time? What about during the day time?		
Inattention	Impaired ability to focus sustain and shift attention	230	8.7%*
Drowsiness	Disturbance of consciousness, that is either being sleepy, or awake but unaware of their surroundings	142	6.5%
	Is the subject drowsy now?		
Delirium judgment	Could a physical illness (not drugs or alcohol intoxication) be sufficient explanation for the subject's mental or psychiatric symptoms (e.g. delirious due to acute infection)?	34	1.6%

*Question comes from prevalence assessment “Ascertain”, denominator = 2640, all other questions from History and Aetiology Schedule (“Informant”).

A symptom was scored as ‘present’ if a positive response was given to one or more question in the cluster.

Inattention assessed by examination of participant with serial 7s task or counting backwards from 20.

**Table 2:** Delirium algorithm

Delirium	= Informant reporting [Acute change] + [Fluctuation] + [Inattention and/or drowsiness]
	OR
	= Interviewer judgment: a physical illness … be sufficient explanation for the subject's mental or psychiatric symptoms (e.g. delirious due to acute infection)

### Statistical analyses

The criterion validity of the study-defined delirium algorithm was tested in two ways: (i) hazard for mortality and (ii) odds of a new diagnosis of dementia at two year follow-up. The association between study-defined delirium and mortality was evaluated using Cox proportional hazards models, adjusted by age, sex and prevalent dementia. The association between study-defined delirium and dementia was assessed using logistic regression where the outcome was new dementia at two-year follow-up (“Follow-up”, Figure 1) in the sample known to be dementia-free at baseline, adjusted by age and sex (“Ascertain”, Figure 1). Each delirium symptom was tested for both outcomes, as well as the overall algorithmic diagnosis. Testing the criterion validity of the algorithmic diagnosis also adjusted for interviewers’ rating of current acute illness severity. Post-estimation tests for underlying assumptions included Hosmer-Lemershow goodness-of-fit and Schoenfeld residuals for logistic and proportional hazards models respectively.

## Results

The subsample selected for this analysis included 2197 individuals assessed by both the GMS (participant) and HAS (informant) schedules at the assessment interview (“Ascertain”). Median age was 77 (interquartile range 71-84), and 1403 (64%) were women. In this weighted subsample of the whole baseline cohort, 511 (23%) had prevalent dementia. Table 1 lists the delirium symptom clusters and questions used to ascertain these, along with the prevalence of individual symptoms. Table 3 reports the age and sex characteristics of the sample, number of persons with study-defined delirium and dementia, and incident dementia and mortality at two years.

**Table 3:** Cases of study-defined delirium and dementia, with outcomes at two years later

		**No delirium (n = 2075)**		**Delirium (n = 122)**	
	Age (median, IQR)	76.8	(70.8 – 83.5)	83.2	(75.4 – 87.7)
	Sex (n women, %)	1316	(63)	87	(71)
Dementias at baseline	Cases	425	*21%*	86	*72%*
	Denominator	2065		119	
	Missing	10		3	
Death within two-year interval	Cases	706	*34%*	72	*61%*
	Denominator	2065		119	
	Missing	10		3	
Incident dementia at follow-up two years later	Cases	102	*9%*	9	*45%*
	Denominator	1129		20	
	Missing (not including deaths)	240		27	

Groups described here are from the assessed population, i.e. 20% most cognitively impaired at screen, plus random sample of remainder.

Delirium, as defined through study algorithm.

IQR interquartile range.

Dementia diagnoses from AGECAT.

Table 4 shows the results of the Cox proportional hazards survival analyses, adjusted by age, sex and prevalent dementia. In this weighted subsample, each delirium symptom was independently associated with higher mortality. This was also the case for the algorithmic diagnosis, even after adjustment for acute illness severity (HR 1.28, 95% CI 1.03 to 1.60). A similar risk for study-defined subsyndromal delirium was apparent (HR 1.41, 95% CI 1.23 to 1.62).

**Table 4:** Survival models for study-defined delirium

	**N**	**Missing data**	**HR**	**LCI**	**UCI**	**P**
Delirium symptom clusters						
Inattention	2637	3	1.36	1.16	1.58	<0.01
Acute change	2184	13	1.57	1.33	1.85	<0.01
Fluctuation	2184	13	1.40	1.21	1.62	<0.01
Drowsiness	2184	13	1.31	1.08	1.57	<0.01
Judged delirium*	2184	13	1.92	1.35	2.74	<0.01
Delirium: final model	2159	38				
Delirium			1.28	1.03	1.60	0.03
Dementia			1.83	1.63	2.06	<0.01
Age (per year)			1.08	1.08	1.09	<0.01
Sex (women versus men)			0.68	0.61	0.75	<0.01
Illness severity						
None			[Ref]			
Mild			1.47	1.15	1.88	<0.01
Moderate			1.52	1.10	2.12	<0.01
Severe			3.14	2.23	4.42	<0.01
Subsyndromal delirium: final model	2159	38				
Sybsyndromal delirium			1.41	1.23	1.62	<0.01
Dementia			1.62	1.42	1.85	<0.01
Age (per year)			1.08	1.07	1.09	<0.01
Sex (women versus men)			0.67	0.61	0.75	<0.01
Illness severity						
None			[Ref]			
Mild			1.32	1.03	1.70	0.03
Moderate			1.50	1.10	2.06	0.01
Severe			2.94	2.10	4.12	<0.01

HR hazard ratio, LCI UCI 95% lower and upper confidence intervals respectively.

This table shows Cox proportional hazard models for death.

“Delirium symptom clusters” shows individual symptom clusters, and their association with mortality (adjusted for age, sex, baseline dementia and illness severity), i.e. the independent effects of each symptom cluster.

*‘Judged delirium’ refers to the overall impression of the interviewer that a participant had delirium.

“Delirium: final model” refers to the full model for full syndromal delirium and the same adjusted covariates.

“Subsyndromal delirium: final model” describes the full model for subsyndromal delirium and the same adjusted covariates.

**Missing data.** These arise through non-responses in interviewed participants or respondents. Estimated models are based on complete cases, i.e. where all covariates are complete for the sample.

**Figure 2:**
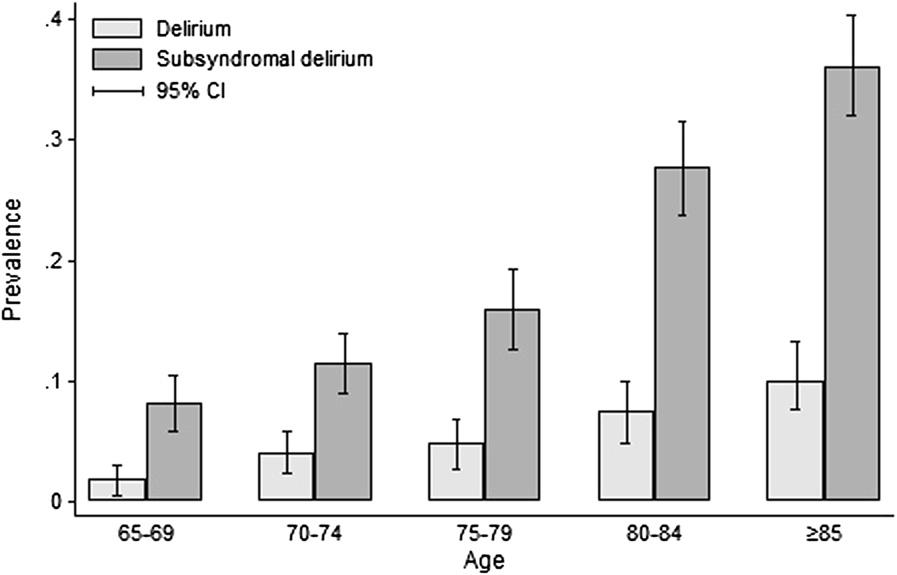
**Prevalence of study-defined delirium and subsyndromal delirium, by age group.** Bar chart showing estimated age-specific prevalence of the algorithm diagnosis of delirium (grey) and subsyndromal delirium (dark grey) as a proportion of the assessed subsample. Upper and lower bars show 95% confidence intervals.

Table 5 gives the results of the logistic regression analyses assessing the odds of a dementia diagnosis at two year follow-up, adjusted by age and sex. In this weighted subsample, all delirium symptoms were associated with odds ratios greater than 1.0, but this was only statistically significant for acute change, fluctuation and drowsiness. The algorithmic diagnosis was significantly associated with a two year dementia diagnosis (OR 8.82, 95% CI 2.76 to 28.2). The estimate for study-defined subsyndromal delirium was half that of full syndromal delirium (OR 4.31, 95% CI 2.41 to 7.73).The estimated age-specific period prevalence of the algorithmic diagnosis of delirium is given in Figure 2. The overall period prevalence in this subsample of enriched for cognitive impairment is estimated at 5.6% (95% CI 4.6 to 6.5). Age-specific prevalence increases with age from 1.8% in the 65-69 year age group to 10.1% in the ≥85 age group (p < 0.01 for trend). For study-defined subsyndromal delirium, age-specific period prevalence ranged from 8.2% (65-69 years) to 36.1% (≥85 years). In persons with prevalent dementia, 16.8% (95% CI 13.6 to 20.1%) had superimposed delirium.

**Table 5:** Logistic models for two year dementia

	N	**Missing data**	**OR**	**LCI**	**UCI**	**P**
Delirium symptom clusters						
Inattention	1347	37	1.90	0.77	4.69	0.16
Acute change	1149	235	7.63	3.47	16.75	<0.01
Fluctuation	1347	37	6.84	3.67	12.77	<0.01
Drowsiness	1347	37	4.83	2.50	9.35	<0.01
Judged delirium*	1149	235	4.44	0.78	25.26	0.09
Delirium: final model	1140	244				
Delirium			8.82	2.76	28.2	<0.01
Age (per year)			1.11	1.08	1.14	<0.01
Sex (women versus men)			0.96	0.61	1.50	0.85
Illness severity						
None			[Ref]			
Mild			1.66	0.57	4.79	0.35
Moderate			1.41	0.31	6.37	0.66
Severe			(omitted)**			
Subsyndromal delirium: final model	1140	244				
Subsyndromal delirium			4.31	2.41	7.73	<0.01
Age (per year)			1.10	1.07	1.14	<0.01
Sex (women versus men)			0.94	0.60	1.47	0.78
Illness severity						
None			[Ref]			
Mild			1.02	0.35	2.95	0.98
Moderate			1.54	0.41	5.77	0.52
Severe			(omitted)**			

OR odds ratio, LCI UCI 95% lower and upper confidence intervals respectively.

This table shows logistic regression models for dementia at two year follow-up.

“Delirium symptom clusters” shows individual symptom clusters, and their association with dementia (adjusted for age, sex, baseline dementia and illness severity), i.e. the independent effects of each symptom cluster.

*‘Judged delirium’ refers to the overall impression of the interviewer that a participant had delirium.

“Delirium: final model” refers to the full model for full syndromal delirium and the same adjusted covariates.

“Subsyndromal delirium: final model” describes the full model for subsyndromal delirium and the same adjusted covariates.

**The effect of severe illness was not estimated due to no participants being assigned to this category.

**Missing data.** These arise through non-responses in interviewed participants or respondents. There were also losses to follow-up that did not include death (n = 267). Estimated models are based on complete cases, i.e. where all covariates are complete for the sample.

## Discussion

These results demonstrate the possibility of constructing an algorithmic diagnosis for delirium within a population-based framework using data from the GMS schedule including self and informant reported responses. This algorithm has criterion validity for mortality and dementia risk. These are the first population-based analyses able to account for both illness severity and prevalent dementia, suggesting that our study-defined delirium has a deleterious effect on mortality and dementia risk beyond that expected from precipitating and predisposing factors alone. These findings also highlight the importance of age in the prevalence of delirium with the highest prevalence in the oldest-old group (i.e. ≥85 years).

The strengths of this study derive from its large population-based sample size and availability of serial cognitive assessments in relation to incident dementia. The major limitation is that the algorithm was not validated with concurrent clinical diagnosis of delirium. Furthermore, the period over which informants were asked to comment on delirium symptoms was not strictly defined (‘in recent weeks and months’) and may be overstated due to recall bias. The lack of assessments by clinicians also limits the precision of the data. Though dementia as a binary predisposing factor in delirium is a well-established construct, delirium could be associated with mild or minimal dementia-type states not captured by the AGECAT diagnosis. Analyses taking the continuum of cognition into account would address this concern to some extent, though there is real difficulty in disentangling such closely associated phenomena, both in clinical and epidemiological studies. Nonetheless, this does not detract from the prognostic significance of delirium symptoms for future dementia. As with any observational study, both over-adjustment and residual confounding remain considerations. It is recognized that the effect of age on total dementia risk is so large that it overshadows many of other associations under investigation (particularly modifiable risk factors) [17,18]. This has been specifically observed in previous CFAS analyses [19,20], and therefore it is not clear which co-morbidities, if any, should also be included in dementia risk models. Finally, though the CFAS sample was population-representative in 1991, the age-specific prevalence of dementia is lower in 2011 [21] and so secular trends may constrain the accuracy of the delirium prevalence estimates.

The estimated age-specific population prevalence of study-defined delirium is lower than the only other estimate available (from GERDA), even though diagnoses included information from community medical records [13]. Previously, the only population-based cohort to have assessed a delirium measure in relation to adverse outcomes is the Vantaa 85+ study [22,23]. In Vantaa 85+, delirium history was assessed at each interview by a neurologist with access to an informant and medical records, amounting to an estimate of period prevalence for the intervening 2-3 years between waves. The present analysis is four times bigger (CFAS = 2197 versus Vantaa = 553). Though medical records were not available here, the advantage in CFAS is the possibility of accounting for illness severity, even though this assessment was subjective. The point estimates for mortality (CFAS HR 1.55 (when unadjusted by illness severity) versus Vantaa HR 1.61) and two-year dementia risk (CFAS OR 8.82 versus Vantaa OR 8.65) are effectively the same. Though delirium diagnoses were derived through different approaches, this suggests the core features of inattention, altered arousal (here characterized as ‘drowsiness’) and acute fluctuations in cognitive function represent an adverse state for future outcomes regardless of the exact methods for operationalizing the syndrome.

## Conclusions

These data add to the small literature on the population-based epidemiology of delirium. Delirium, as defined in this study, is associated with increased dementia, strengthening the argument that interventions for delirium may have an impact on the burden of cognitive impairment. Nonetheless, the core elements of the delirium-dementia relationship still require further exploration (e.g. if the key factors responsible for this association relate to etiology, duration, severity or some combination), particularly in the general population and in other cohort studies [18]. At the least, these findings indicate that it is possible to identify population samples with delirium and subsyndromal delirium at higher risk for dementia.

### Endnote

^a^In other CFAS publications, the stages: S0; A0; H0; C2 are described here as “Screen”; “Ascertainment”; “Informant”; “Follow-up” respectively.

## Competing interests

The authors declare that they have no competing interests.

## Authors’ contributions

DD and JC devised the algorithm. DD undertook the statistical analysis with assistance from BS and FM. LB conducted and co-ordinated the fieldwork for CFAS. AM and DM provided expertise on syndromal and subsyndromal delirium phenomenology. CB contributed to the conception and design of the study, and is a principal investigator on CFAS, along with FM. All authors read and approved the final manuscript.

## Pre-publication history

The pre-publication history for this paper can be accessed here:

http://www.biomedcentral.com/1471-2318/14/87/prepub
